# Effects of fatiguing unilateral plantar flexions on corticospinal and transcallosal inhibition in the primary motor hand area

**DOI:** 10.1186/s40101-015-0042-x

**Published:** 2015-02-24

**Authors:** Ryouta Matsuura, Toru Ogata

**Affiliations:** Laboratory of Kinesiology, Living and Health Sciences Education, Specialized Subject Fields of Education, Graduate School of Education, Joetsu University of Education, 1 Yamayashiki-machi, Joetsu, 943-8512 Japan; Department of Rehabilitation for Movement Functions, Research Institute, National Rehabilitation Center for Persons with Disabilities, 4-1 Namiki, Tokorozawa, 359-8555 Japan

**Keywords:** Muscle fatigue, Transcranial magnetic stimulation, Interlimb, Laterality, Interhemispheric inhibition

## Abstract

**Background:**

Corticospinal excitability of the primary motor cortex (M1) representing the hand muscle is depressed by bilateral lower limb muscle fatigue. The effects of fatiguing unilateral lower limb contraction on corticospinal excitability and transcallosal inhibition in the M1 hand areas remain unclear. The purpose of this study was to determine the effects of fatiguing unilateral plantar flexions on corticospinal excitability in the M1 hand areas and transcallosal inhibition originated from the M1 hand area contralateral to the fatigued ankle.

**Methods:**

Ten healthy volunteers (26.2 ± 3.8 years) participated in the study. Using transcranial magnetic stimulation, we examined motor evoked potentials (MEPs) and interhemispheric inhibition (IHI) recorded from resting first dorsal interosseous (FDI) muscles before, immediately after, and 10 min after fatiguing unilateral lower limb muscle contraction, which was consisted of 40 unilateral maximal isometric plantar flexions intermittently with a 2-s contraction followed by 1 s of rest.

**Results:**

We demonstrated no significant changes in MEPs in the FDI muscle ipsilateral to the fatigued ankle and decrease in IHI from the M1 hand area contralateral to the fatigued ankle to the ipsilateral M1 hand area after the fatiguing contraction. MEPs in the FDI muscle contralateral to the fatigued ankle were increased after the fatiguing contraction.

**Conclusions:**

These results suggest that fatiguing unilateral lower limb muscle contraction differently influences corticospinal excitability of the contralateral M1 hand area and IHI from the contralateral M1 hand area to the ipsilateral M1 hand area. Although fatiguing unilateral lower limb muscle contraction increases corticospinal excitability of the ipsilateral M1 hand area, the increased corticospinal excitability is not associated with the decreased IHI.

## Background

Corticospinal excitability is altered during recovery from muscle fatigue. For example, corticospinal excitability is depressed in resting fatigued muscle [1-4]. Reduced corticospinal excitability after muscle fatigue is observed in not only fatigued muscle but also the non-fatigued homonymous muscle [5-7]. Takahashi et al. [7] speculated that depression of short-interval intracortical inhibition (SICI) in the exercised primary motor cortex (M1) [4] is responsible for the reduced corticospinal excitability of the non-exercised M1 after muscle fatigue because the reduced SICI disinhibits activity of transcallosal glutaminergic neurons from the exercised to non-exercised M1, and the disinhibition increases transcallosal inhibition from the exercised to non-exercised M1. Their conclusion was that fatiguing unilateral muscle contraction has separate effects on corticospinal excitability of the exercised M1 and transcallosal inhibition originated from the exercised M1. Indeed, this conclusion is partly supported by data from a study [8] which showed that a phasic fatiguing pinch grip task have separate effects on corticospinal excitability for the exercised muscle and transcallosal inhibition from the exercised to non-exercised M1.

Muscle fatigue also decreases corticospinal excitability for a distinct segment muscle. Corticospinal excitability for the right upper limb is reduced during recovery from fatiguing bilateral leg press [9]. However, no study has investigated whether transcallosal inhibition between M1s representing upper limb muscles is altered after fatiguing lower limb contraction. If fatiguing lower limb contraction has separate effects on the activity of corticospinal and transcallosal neurons in the M1 upper limb area as is the case with the study [8] focused on fatigued muscle, corticospinal excitability for the upper limb and transcallosal inhibition between M1 upper limb areas decrease and increase during recovery from lower limb muscle fatigue, respectively. Consequently, the increased transcallosal inhibition from one to the opposite M1 may depress excitability of the opposite M1 [7]. Note that the decreased corticospinal excitability for the right upper limb after fatiguing ‘bilateral’ lower limb contraction [9] may be due to reduced excitability of the left M1 hand area, increased transcallosal inhibition from the right to left M1 hand area, or both. Therefore, the present study applied ‘unilateral’ lower limb contraction as a fatigue task to specify the laterality of effects of fatiguing lower limb contraction on corticospinal and transcallosal pathways from the M1 upper limb area. We hypothesized that fatiguing unilateral lower limb contraction will have separate effects on corticospinal excitability of the M1 hand area contralateral (M1_contra-H_) to the fatigued lower limb and transcallosal inhibition from the M1_contra-H_ to M1 hand area ipsilateral (M1_ipsi-H_) to the fatigued lower limb (i.e., decrease and increase, respectively) and subsequently decrease corticospinal excitability of the M1_ipsi-H_.

To test our hypothesis, we used transcranial magnetic stimulation (TMS) to measure motor evoked potentials (MEPs) in the upper limb muscles before and after fatiguing unilateral lower limb contraction. To assess transcallosal inhibition from the M1_contra-H_ to M1_ipsi-H_ (CtoI), paired-pulse interhemispheric inhibition (IHI) was used. Fatiguing handgrip exercise decreases the ankle plantar-flexor maximal voluntary contraction (MVC) due to central factor [10]. This suggests that there is neural interaction between plantar flexor muscles and hand muscles. Therefore, the present study investigated the effect of fatiguing unilateral plantar flexion on MEPs in both of the first dorsal interosseous (FDI) muscles.

## Methods

### Subjects

Ten healthy volunteers (26.2 ± 3.8 years old, two females) participated in the study. All participants were right-handed and right-footed. All subjects gave written informed consent, and the experimental procedures were carried out in accordance with the Declaration of Helsinki. The Ethics Committee of Japan’s National Rehabilitation Center for Persons with Disabilities approved the study. The subjects were informed about the experimental procedures but were kept unaware of the precise experimental hypotheses. Handedness and footedness were assessed with the Edinburgh Handedness Inventory [11] and Chapman foot preference inventory [12], respectively.

### Recordings

Electromyographic (EMG) activity was recorded bilaterally from the FDI muscles through surface electrodes (Ag/AgCl; 7 mm diameter) secured to the skin over the belly of each muscle. The ground electrode was placed at the right elbow. EMG signals were amplified and filtered (bandwidth, 15 to 1,000 Hz) with a bioamplifier (AB-611 J, Nihon Kohden, Tokyo, Japan). Isometric ankle plantar flexion torque was measured using a custom-made chair (Senoh Inc., Tokyo, Japan) with two stationary footplates that were connected to servo-controlled torque motors with rotary encoders [13-15]. All signals were stored on a computer with sampling rate of 2 kHz using an analog-digital converter (PowerLab 8/30, ADInstruments, Bella Vista, NSW, Australia) for later off-line analysis (LabChart v7.3.1 for Windows, ADInstruments, Bella Vista, NSW, Australia).

### Experimental protocol

**Figure 1:**
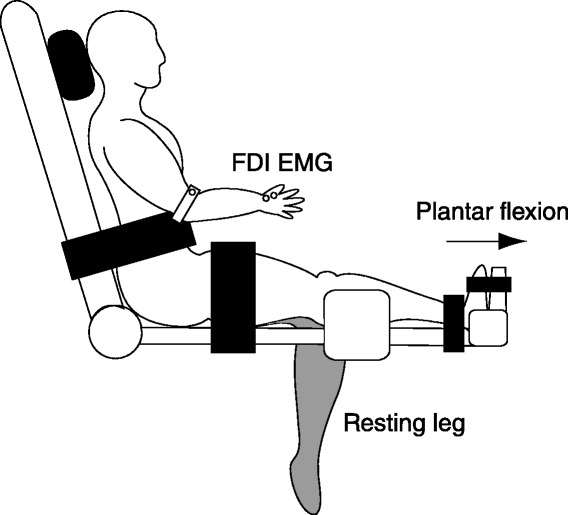
**Schematic illustration of the experimental setup.** Subjects were instructed to performed unilateral maximal isometric plantar flexions as a fatigue task. The exercising ankle was strapped to with footplate with the knee and ankle angles at full extension and a neutral position, respectively. Subjects were asked to keep the non-exercising leg at rest.

Subjects performed unilateral maximal isometric plantar flexions intermittently with a 2-s contraction followed by 1 s of rest and repeated 40 times (i.e., 120 s). The contraction rate was controlled by audio cues provided by the computer. Before the fatigue task, subjects performed three unilateral maximal isometric plantar flexions for 5 s separated by 60 s of rest to avoid fatigue. The highest value with respect to torque from the three trials was considered to be the MVC. An exercising side (i.e., the left or right ankle) was counterbalanced across subjects (left: *n* = 5, right: *n* = 5). During both the fatigue task and MVC trials, subjects were seated in the custom-made chair (Figure 1) with the shoulder and elbow angles semi-flexed, and the knee of the non-exercising leg and hip angles positioned at 90° and 120°, respectively. The exercising ankle was strapped to with footplate with the knee and ankle angles at full extension and a neutral position, respectively. The rotational axis of the footplate was aligned to the center of the ankle joint. Straps were fastened across the subject’s torso and thighs to minimize body movement. During both the fatigue task and MVC trials, standard verbal encouragement and online real-time visual feedback were provided and subjects were asked to keep the non-exercising leg and hand at rest. Before the MVC trials and after the fatigue task, subjects were seated comfortably in a chair with the left and right arms flexed at 90°, forearm pronated, and the wrist restrained by the straps to measure single-pulse MEPs and paired-pulse IHI. TMS measurements were performed before (PRE), immediately (POST_1_), and 10 min after (POST_2_) the fatigue task.

### TMS

For single-pulse MEPs, TMS was performed with a single Magstim 200 stimulator (Magstim Co., Whitland, UK) connected with a figure-eight coil (each 70 mm in diameter). For paired-pulse IHI, TMS was given through two Magstim stimulators connected to two figure-eight coils (each 70 mm in diameter). The coils were placed tangentially to the scalp with the handle pointing postero-laterally at around 45° to the midline with induced current in the cortex flowing posterior to anterior across the motor strip. We determined the optimal position for the activation of the left and right FDI muscles overlying left- and right-hand M1s. To mark the optimal position on the scalp with ink for allowing a re-positioning of the coil throughout the experiment, subjects were asked to wear a swimming cap. TMS measurements included resting motor threshold (RMT, only PRE), single-pulse MEPs in the FDI muscles ipsilateral and contralateral to the fatigued ankle (FDI_ipsi_ and FDI_contra_, respectively), and CtoI IHI. RMT (FDI_ipsi_, 46.8% ± 7.3% of maximal stimulator output (MSO); FDI_contra_, 46.8% ± 6.5% of MSO) was defined as the minimum stimulus intensity required to induce MEPs of at least 0.05 mV peak-to-peak amplitude in five of the ten consecutive trials in the relaxed FDI muscle. In each time point, single-pulse MEPs in the FDI_ipsi_ and FDI_contra_ were recorded in randomized order. Subsequently, paired-pulse IHI was recorded. In some subjects, it was not possible to hold both coils at the optimal positions because of the size of the coil and minor changes of coil positions were required. In these subjects, the stimulus intensities required to obtain RMT and single-pulse MEPs were determined with the coils at the adjusted positions, and same positions were used throughout the experiment.

#### Single-pulse MEPs

At all times, the left and right FDI muscles remained at rest. Ten single-pulse MEPs were recorded in each FDI muscle (i.e., FDI_ipsi_ and FDI_contra_) at stimulation intensity (FDI_ipsi_, 56.5% ± 11.0% of MSO; FDI_contra_, 55.6% ± 7.2% of MSO) necessary to elicit a peak-to-peak amplitude of approximately 0.60 mV. This intensity was maintained unchanged throughout the experiment. TMS pulses were given every 5 s. MEP amplitudes were measured peak to peak and averaged off-line.

#### Paired-pulse IHI

At all times, the left and right FDI muscles remained at rest. IHI was tested after a randomized condition test design reported previously [16]. A suprathreshold conditioning stimulus (CS) was given to the M1_contra-H_ 10 ms before a test stimulus (TS) delivered to the M1_ipsi-H_. The TS was adjusted to produce a MEP of approximately 0.60 mV peak-to-peak amplitude in the FDI_contra_. The CS was set at 110% (53.1% ± 9.4% of MSO) of RMT in the FDI_ipsi_ and elicited an amount of inhibition of 23.3% ± 10.4%. Stimuli were randomly delivered in one set of 20 trials: 10 conditioned and 10 unconditioned. IHI was expressed as the ratio between the mean peak-to-peak MEP amplitude in conditioned versus unconditioned trials.

### Plantar flexion torque

In the fatigue task and MVC trials, mean torque (500 ms around peak torque) was calculated during each contraction. We expressed torque data as a percentage of the MVC value.

### Statistical analysis

Results are presented as means ± standard deviation (SD). For single-pulse MEPs and stimulus intensity, two-way analysis of variance (ANOVA) with repeated measures was performed with time (PRE, POST_1_, and POST_2_) and laterality (FDI_ipsi_ and FDI_contra_) as factors. For paired-pulse IHI, one-way ANOVA with repeated measures was performed with time (PRE, POST_1_, and POST_2_) as factor. All variables were examined using Mendoza’s multisample sphericity test. Whenever the data violated the assumption of sphericity, *p* values based on the Greenhouse-Geisser correction were reported. After ANOVA, Shaffer’s modified sequentially rejective Bonferroni procedure was performed for multiple comparisons. Significance was set at *p* < 0.05.

## Results

### Fatigue task

MVC in plantar flexion was 124.8 ± 32.9 (range: 68.2 to 181.2) N · m. Mean torque was decreased to 60.8% ± 18.1% (range: 25.8% to 83.7%) MVC at the end of fatiguing plantar flexions.

### Single-pulse MEPs

**Figure 2:**
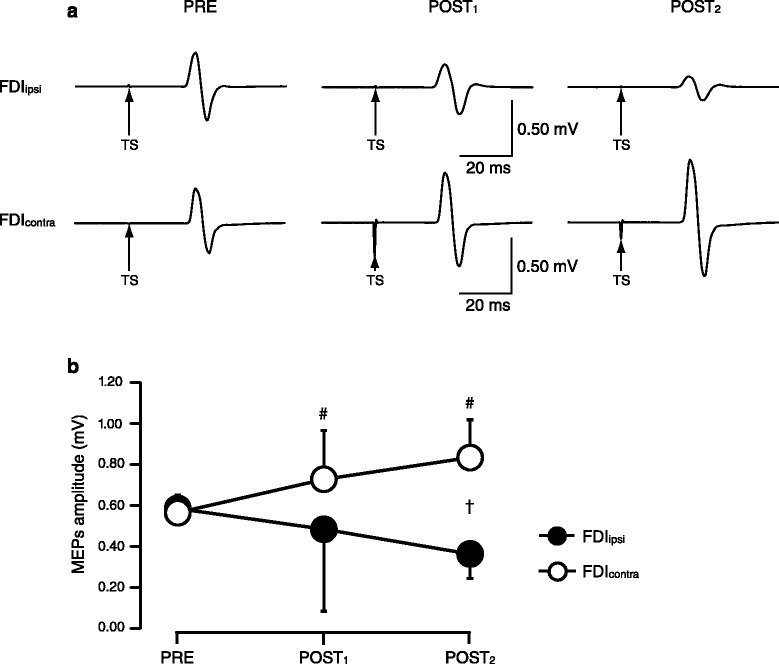
**Single-pulse motor evoked potentials (MEPs) recorded from the first dorsal interosseous (FDI) muscles before (PRE), immediately after (POST**_**1**_ **), and 10 min after (POST**_**2**_ **) fatiguing unilateral plantar flexions. (a)** MEPs in the FDI muscle ipsilateral (FDI_ipsi_, top traces) and contralateral (FDI_contra_, bottom traces) to the fatigued lower limb of a representative subject. Arrows marked with TS indicate the time points of stimulation. Ten traces were average in each set. **(b)** Group data (*n* = 10). The abscissa shows time points of measurements (PRE, POST_1_, and POST_2_). The ordinate shows the magnitude of MEPs. Filled and open circles represent MEPs in the FDI_ipsi_ and FDI_contra_, respectively. ^#^indicates statistical significance from PRE in the FDI_contra_ (*p* < 0.05). ^†^indicates statistical significance between the FDI_ipsi_ and FDI_contra_ (*p* < 0.05). Error bars indicate SD.

Figure 2a illustrates single-pulse MEPs in the FDI_ipsi_ and FDI_contra_ recorded in a single subject at PRE, POST_1_, and POST_2_. Repeated measures ANOVA showed a significant interaction between time and laterality (*F*_(2,18)_ = 8.0, *p* = 0.003; Figure 2b). Single-pulse MEPs in the FDI_ipsi_ decreased with time but the decrease did not reach statistical significance (PRE, 0.59 ± 0.06 mV; POST_1_, 0.49 ± 0.40 mV; POST_2_, 0.37 ± 0.12 mV; *F*_(1.25,14)_ = 11.3, *p* = 0.16). In the FDI_contra_, single-pulse MEPs significantly increased with time (PRE, 0.57 ± 0.08 mV; POST_1_, 0.73 ± 0.24 mV; POST_2_, 0.84 ± 0.18 mV; *F*_(2,14)_ = 10.3, *p* = 0.001) and were significantly larger at POST_1_ (*p* = 0.04) and POST_2_ (*p* < 0.001) compared to PRE. MEPs in the FDI_contra_ were significantly larger than those in the FDI_ipsi_ at POST_2_ (*F*_(1,9)_ = 34.2, *p* < 0.001). At PRE, single-pulse MEPs were not significantly different between the FDI_ipsi_ and the FDI_contra_ (*F*_(1,7)_ = 1.0, *p* = 0.34).

### Paired-pulse IHI

**Figure 3:**
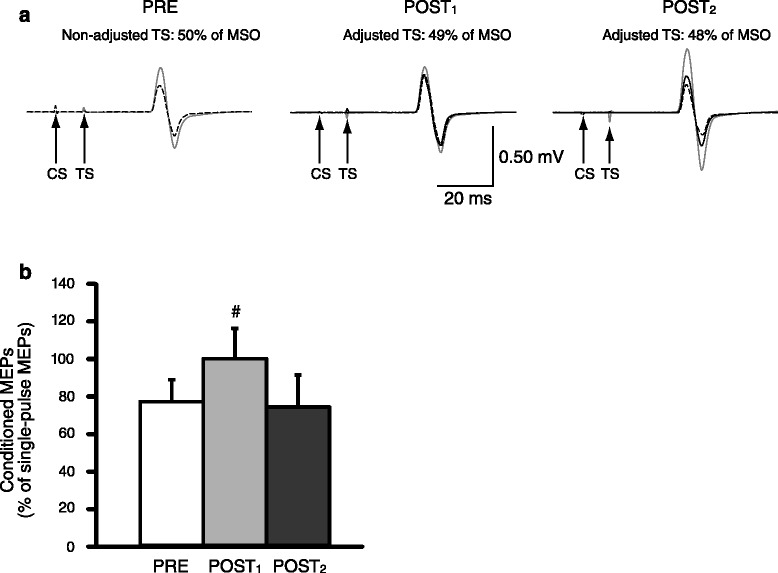
**Interhemispheric inhibition (IHI) from the M1 hand area contralateral (M1**_**contra-H**_ **) to that ipsilateral (M1**_**ipsi-H**_ **) to the fatigued lower limb.** **(a)** IHI from the M1_contra-H_ to M1_ipsi-H_ (10 min) recorded from the first dorsal interosseous muscle of a representative subject before (PRE, left column), immediately after (POST_1_, middle column), and 10 min after (POST_2_, right column) fatiguing unilateral plantar flexions. Solid lines are single-pulse motor evoked potentials (MEPs) elicited by adjusted test stimulus (TS) intensity; gray lines are single-pulse MEPs elicited by non-adjusted TS intensity; and dashed lines are conditioned MEPs. Arrows indicate the time points at the TS and conditioning stimulus (CS) are delivered. Ten traces were average in each set. Note that the adjusted TS intensities (% of maximal stimulator output (MSO)) decreased with time. **(b)** Group data (*n* = 10). The abscissa shows time points of measurements (PRE, POST_1_, and POST_2_). The ordinate shows the magnitude of IHI, in which the size of the conditioned MEPs is expressed as a percentage of the size of the adjusted single-pulse MEPs. ^#^indicates statistical significance from PRE (*p* < 0.05). Error bars indicate SD.

Figure 3a illustrates changes in CtoI IHI recorded in a single subject at PRE, POST_1_, and POST_2_. Repeated measures ANOVA showed a significant effect of time on CtoI IHI (PRE, 76.7 ± 10.4%; POST_1_, 99.6 ± 18.3%; POST_2_, 73.8 ± 15.6%; *F*_(2,18)_ = 12.5, *p* < 0.001; Figure 3b). CtoI IHI was decreased at POST_1_ compared to PRE (*p* = 0.01). There was no significant difference between CtoI IHI at PRE and POST_2_ (*p* = 0.49). The absolute amplitude of unconditioned MEPs were maintained throughout the experiment (PRE, 0.57 ± 0.08 mV; POST_1_, 0.55 ± 0.07 mV; POST_2_, 0.58 ± 0.08 mV; *F*_(1.13,11.88)_ = 1.7, *p* = 0.22). The adjusted TS intensities significantly decreased with time (PRE, 117.0 ± 7.0% of RMT; POST_1_, 114.8% ± 6.7% of RMT; POST_2_, 113.8% ± 6.1% of RMT; *F*_(2,18)_ = 19.2, *p* < 0.001) and were significantly smaller at POST_1_ (*p* = 0.002) and POST_2_ (*p* < 0.001) compared to PRE.

## Discussion

We found separate effect of fatiguing unilateral lower limb contraction on corticospinal excitability of the M1_contra-H_ and CtoI transcallosal inhibition. However, contrary to our expectations, corticospinal excitability of the M1_contra-H_ was not significantly reduced and CtoI transcallosal inhibition was reduced immediately after the fatigue task. Surprisingly, corticospinal excitability of the M1_ipsi-H_ increased rather than decreased after the fatigue task. This increase of corticospinal excitability of the M1_ipsi-H_ was maintained until 10 min after the fatigue task but the decrease of CtoI transcallosal inhibition was not.

### Corticospinal excitability of M1 hand areas after fatiguing unilateral plantar flexions

Inconsistent with results of the previous study [9], MEPs in the FDI_ipsi_ was not significantly reduced for 10 min after the fatigue task. This inconsistency may be explained by the differences in muscle groups and the amount of muscle mass used in the fatigue task. In the previous study [9], subjects performed dynamic leg press in fatigue task, but plantar flexors was selected as fatiguing muscle in the present study. Dynamic leg press involves the tibialis anterior muscle to which the strongest corticospinal projections are in the lower limb [17]. Therefore, cortical contribution to fatigue task might be greater in the previous study [9] than in the present study and the greater contribution might strongly influence central factors associated with decrease in corticospinal excitability of M1 hand area. Additionally, the amount of muscle mass used in fatigue task was greater in the previous study [9] than in the present study (i.e., bilateral leg press vs. unilateral plantar flexion). The greater amount of muscle mass must result in increase in the amount fatigue created within exercised muscles, resulting in the central changes occurring in reaction to the peripheral changes [18]. The central changes due to peripheral changes might be related to the decrease in corticospinal excitability for a distinct segment muscle after muscle fatigue. Alternatively, difference in movement mode (i.e., phasic vs. isometric) may also be responsible for the inconsistency. Corticospinal excitability for the exercised muscle is facilitated during repeated muscle contraction compared to sustained contraction [19] and decrease in corticospinal excitability for the exercised muscle is greater in repeated contraction than in sustained contraction after muscle contraction [20]. This suggests that facilitation of corticospinal excitability during muscle contraction is responsible for the decrease in corticospinal excitability after muscle contraction. It has been shown that corticospinal excitability of the M1 arm area is facilitated by phasic ankle contraction compared to isometric contraction [21]. Thus, it is likely that more facilitation of corticospinal excitability for the hand muscles during phasic leg press than isometric plantar flexion resulted in more decrease in corticospinal excitability after muscle fatigue.

Contrary to the FDI_ipsi_, MEPs in the FDI_contra_ significantly increased for 10 min after fatiguing unilateral plantar flexions. The increased MEPs may be not associated with the changes in CtoI IHI after the fatigue task because CtoI IHI decreased only immediately after the fatigue task. Since facilitation of corticospinal excitability for a distinct segment muscle has been observed in not only the muscle ipsilateral to the exercised muscle but also the contralateral one during high-intensity muscle contraction [22], MEPs in the FDI_contra_ should not have been increased after the fatigue task if facilitation of corticospinal excitability for a distinct segment muscle was responsible for the decreased excitability after muscle fatigue. Tazoe et al. [23] demonstrated that only MEPs but not cervicomedullary MEPs of a distinct segment muscle were facilitated during a fatiguing contraction and suggested that the facilitation of a distinct segment muscle depends on the activity of upstream of the M1 during a fatiguing contraction. Therefore, it is likely that the increased MEPs in the FDI_contra_ were not induced by only input from upstream of the M1 during the fatigue task. Since CtoI IHI was reduced immediately after the fatigue task, it is possible that CtoI IHI was inhibited during the fatigue task. Indeed, IHI from the left to right M1 upper limb area is decreased during right dorsiflexion at 75% MVC [22]. The increased corticospinal excitability for the FDI_contra_ is likely to be dependent on interaction between increased activation of corticospinal pathways by input from upstream of the M1 and decreased CtoI IHI. This hypothesis is in accordance with results by Avanzino et al. [24] showing that interaction between the reduction of IHI from the left to right M1 by 10-h right-arm immobilization and increased activation of the right M1 by left-arm overuse induced right M1 plasticity (i.e., increase in MEPs). This indicates possibility that IHI in M1 hand areas is bidirectionally decreased during fatiguing bilateral lower-limb contraction. If so, MEPs in the FDI_ipsi_ and FDI_contra_ will increase due to the interaction between reduced IHI and increased M1 activity after the fatiguing bilateral lower-limb contraction. However, this expectation is contrary to results by Takahashi et al. [9] that observed a reduction of MEPs of the right FDI muscle after fatiguing bilateral leg press. This discrepancy may be explained by changes in SICI in the M1 hand area after the fatiguing bilateral contractions. Since fatiguing bilateral lower limb contractions reduced SICI in the left M1 hand area [7], reduction of SICI might be elicited in both left and right M1 hand areas. As a result of this reduction of SICI, IHI might be bidirectionally increased [25] and an increase of MEPs in the FDI muscle might not be elicited after fatiguing bilateral lower limb contractions. Nevertheless, SICI in the M1 hand area was not measured in the present study. Further studies will be required to elucidate precise mechanisms responsible for the increase of corticospinal excitability for the FDI_contra_ after fatiguing unilateral plantar flexions.

### Interhemispheric inhibition from M1 hand area contralateral to the fatigued ankle after fatiguing unilateral plantar flexions

The fatiguing unilateral plantar flexions decreased IHI from M1_contra-H_ to M1_ipsi-H_. Decreased CtoI IHI after the fatigue task is inconsistent with results by Edgley and Winter [8], who found separate effects of corticospinal excitability of exercised M1 and IHI from the exercised to non-exercised M1. The present study investigated the effect of fatiguing unilateral ‘ankle’ contractions on IHI between M1 hand areas but the previous study [8] investigated the effect of fatiguing unilateral ‘hand’ contractions on IHI between M1 hand areas. This methodological difference may be associated with the inconsistency between results by the present study and previous study. During unilateral muscle contraction, IHI targeting to the M1 upper-limb area is more decreased when homonymous upper-limb muscle rather than lower-limb muscle is contracted [22]. Although no study has investigated the effects of the magnitude of IHI during unilateral muscle contraction on resting IHI after the contraction, differences in activity of transcallosal pathways targeting to M1 hand area between fatiguing unilateral hand and ankle tasks may influence changes in IHI after fatiguing unilateral muscle contraction.

### Limitations

The present study investigated effects of unilateral fatiguing plantar flexions on MEPs in the FDI muscles. MEP is a useful index to estimate corticospinal excitability but reflects excitability of both the cortical and spinal circuits. Therefore, the changes in MEPs in the present study might not necessarily be due to cortical changes. Brasil-Neto et al. [1] found that in a fatigued muscle MEPs by TMS was reduced after muscle fatigue but H-reflexes and MEPs by transcranial electric stimulation did not change, suggesting that MEPs decreased by muscle fatigue are due to cortical changes. Although these results are not absolutely applied to non-fatigued muscles, it is likely that decreased MEPs in the FDI_ipsi_ and FDI_contra_ muscles were mainly due to cortical changes.

Corticospinal excitability of the M1_contra-H_ decreased with time although the decrease did not reach statistical significance (refer to Figure 2a, FDI_ipsi_). Therefore, the CS intensity applied to the M1_contra-H_ should have been adjusted to compensate for the reduction in corticospinal excitability in the M1_contra-H_ in each subject during the CtoI IHI measurement. If the CS intensity was adjusted after the fatigue task, IHI may have increased at POST_1_ and POST_2_. Even though IHI increased after the fatigue task, conclusions that fatiguing unilateral plantar flexions differently influence corticospinal excitability and transcallosal inhibition in the M1_contra-H_ do not change. Nevertheless, it should be noted that IHI by the non-adjusted CS intensity was similar to that by the adjusted CS intensity to compensate for the decreased corticospinal excitability at rest [26].

Finally, the exercising ankle was counterbalanced in the present study. The nondominant-hand muscle contraction increased corticospinal excitability of the contralateral M1 compared to the dominant hand [27] and the left M1 is controlled by less inhibitory tone than the right M1 in right-handers [28]. Thus, laterality has been observed in the M1 hand area. It is possible that this laterality influenced corticospinal excitability and transcallosal inhibition in the present study. The two-way ANOVA did not reveal interaction between the exercising side and TMS indices (MEPs in the FDI_ipsi_, *F*_(2,8)_ = 0.7, *p* = 0.51; MEPs in the FDI_contra_, *F*_(2,8)_ = 0.2, *p* = 0.84; CtoI IHI, *F*_(2,8)_ = 1.3, *p* = 0.32). These results indicate that laterality of CtoI IHI might not be produced after fatiguing unilateral plantar flexions. Since a simple unilateral motor task did not result in laterality of IHI targeting to the ipsilateral M1 [29], the simple fatigue tasks may produce no laterality of CtoI IHI. However, further studies will be required to elucidate laterality of IHI between the M1 hand areas after fatiguing unilateral lower limb contractions.

### Functional significance

It has been widely known that decrease in IHI due to unilateral dysfunction such as stroke leads to facilitation of the cortical excitability in the opposite (i.e., unaffected) hemisphere [30,31]. The facilitation in the unaffected hemisphere increases not only corticospinal excitability but also IHI from the unaffected hemisphere to the affected hemisphere and results in unbalance of excitability in M1s. The unbalance of excitability in M1s is responsible for impairment of recovery of motor functions [32]. Repetitive TMS (rTMS) with a low frequency has been shown to decrease cortical excitability of the stimulated hemisphere [26,33-36] and IHI from the stimulated to the non-stimulated hemisphere [26,34], and increase cortical excitability of the non-stimulated hemisphere [34,35]. Furthermore, rTMS over the M1 improve ipsilateral simple finger movements in healthy subjects [37]. These studies suggest that rTMS over the M1 is a useful technique to improve unbalance of excitability in M1s and ipsilateral motor function. The findings reported here on the increase in MEPs and temporary decrease in IHI are partly similar to these findings with rTMS over the M1 although the facilitation of MEPs in the FDI_contra_ might not result from decrease in IHI. Therefore, fatiguing unilateral ankle contraction may become a novel technique to improve unbalance of excitability in M1 hand areas and ipsilateral hand motor function.

## Conclusions

The present study identifies that fatiguing unilateral plantar flexions differently influence corticospinal excitability of the M1 hand area contralateral to the fatigued ankle and transcallosal inhibition originated from that area. Furthermore, fatiguing unilateral plantar flexions increase corticospinal excitability of the M1 hand area ipsilateral to the fatigued ankle.

## Abbreviations

ANOVA: analysis of variance
CS: conditioning stimulus
CtoI: from the M1_contra-H_ to M1_ipsi-H_
EMG: electromyographic
FDI: first dorsal interosseous
FDI_contra_: FDI contralateral to the fatigued ankle
FDI_ipsi_: FDI ipsilateral to the fatigued ankle
IHI: interhemispheric inhibition
M1: primary motor cortex
M1_contra-H_: M1 hand area contralateral to the fatigued lower limb
M1_ipsi-H_: M1 hand area ipsilateral to the fatigued lower limb
MEP: motor evoked potential
MVC: maximal voluntary contraction
POST_1_: immediately after the fatigue task
POST_2_: 10 min after the fatigue task
PRE: before the fatigue task
RMT: resting motor threshold
rTMS: repetitive TMS
SD: standard deviation
SICI: short-interval intracortical inhibition
TMS: transcranial magnetic stimulation
TS: test stimulus
